# Predicting Odor Pleasantness with an Electronic Nose

**DOI:** 10.1371/journal.pcbi.1000740

**Published:** 2010-04-15

**Authors:** Rafi Haddad, Abebe Medhanie, Yehudah Roth, David Harel, Noam Sobel

**Affiliations:** 1 Department of Neurobiology, Weizmann Institute of Science, Rehovot, Israel; 2 Department of Otolaryngology-Head and Neck Surgery, Edith Wolfson Medical Center, Holon, Israel; 3 Department of Computer Science and Applied Mathematics, Weizmann Institute of Science, Rehovot, Israel; Université Paris Descartes, Centre National de la Recherche Scientifique, France

## Abstract

A primary goal for artificial nose (eNose) technology is to report perceptual qualities of novel odors. Currently, however, eNoses primarily detect and discriminate between odorants they previously “learned”. We tuned an eNose to human odor pleasantness estimates. We then used the eNose to predict the pleasantness of novel odorants, and tested these predictions in naïve subjects who had not participated in the tuning procedure. We found that our apparatus generated odorant pleasantness ratings with above 80% similarity to average human ratings, and with above 90% accuracy at discriminating between categorically pleasant or unpleasant odorants. Similar results were obtained in two cultures, native Israeli and native Ethiopian, without retuning of the apparatus. These findings suggest that unlike in vision and audition, in olfaction there is a systematic predictable link between stimulus structure and stimulus pleasantness. This goes in contrast to the popular notion that odorant pleasantness is completely subjective, and may provide a new method for odor screening and environmental monitoring, as well as a critical building block for digital transmission of smell.

## Author Summary

Electronic noses (eNoses) are devices aimed at mimicking animal noses. Typically, these devices contain a set of sensors that generate a pattern representing an odor. Application of eNoses entails first “training” the eNose to a particular odor, and once the eNose has “learned”, it can then be used to detect and identify this odor. Using this approach, eNoses have been tested in applications ranging from disease diagnosis to space-ship interior environmental monitoring. However, in contrast to animal noses, eNoses have not been used to generate information on novel odors they hadn't learned. Here, rather than train an eNose on particular odorants, we trained an eNose to the perceptual axis of *odorant pleasantness*. We found that this eNose was then able to generalize and rate the pleasantness of novel odors it never smelled before, and that these ratings were about 80% similar to those of naïve human raters who had not participated in the eNose training phase. Furthermore, the results replicated across cultures without retraining of the device. This result contrasts the popular notion that odorant pleasantness is completely subjective, and may allow for numerous applications, such as an environmental monitor that would warn of malodor regardless of its source.

## Introduction

Dravnieks envisioned an artificial (or electronic) nose as “an instrument that would inspect samples of odorous air and report the intensity and quality of an odor without the intervention of a human nose” [1]. Although eNoses have since been developed [2]–[10], and serve in tasks of odor detection and discrimination [7], [11]–[13], they are rarely used for reporting odor quality.

The main component of an eNose is an array of non-specific chemical sensors. An odor analyte stimulates many of the sensors in the array and elicits a characteristic response pattern. The sensors inside eNoses can be made of a variety of technologies, but in all cases a certain physical property is measured and a set of signals is generated. The stages of the recognition process are similar to those of biological olfaction, where a sensor type responds to more than one odorant and one odorant type activates more than one sensor. Together, the set of activated sensors and their signals characterize the odor (sometimes refered as an odor fingerprint). Thus, an important difference between eNoses and analyte detectors such as gas chromatographs, is that whereas the latter are aimed at identifying the components that contribute to an odor, eNoses can be used to identify, as a whole, the mixture of components that together form an odor.

Despite the promise of an artificial system that may substitute for olfaction, few efforts have been made to use eNoses in tasks that go beyond detection and discrimination. A notable exception are the efforts to develop eNoses for medical diagnosis (reviewed in [14] and [15]). In such efforts eNoses were used to identify the disease as a whole, rather than particular analytes that relate to it. In a previous effort from our lab, we used an eNose to predict the receptive range of olfactory receptor neurons [16], suggesting that an eNose can capture the odor attributes relevant to biological receptors. Here we set out to ask whether eNose measurements can similarly be linked to olfactory perception. This effort, however, may be more complicated than linking eNose output to receptor response [16], because perception is governed not only by stimulus structure [17], but also by higher-order mechanisms such as experience and learning [18].

eNose output has been linked to some aspects of perception such as odor intensity [19], and discreet perceptual odor features such as *minty* and *floral* [20]. An alternative approach we explore here is to focus on perceptual axes. Several lines of evidence suggest that the primary perceptual axis of human olfaction is odorant pleasantness [17], [21]–[27]. Furthermore, psychophysical evidence suggested that odorant pleasantness is reflected in part in the physicochemical structure of odorant molecules [17]. With this link in mind, we set out to test the hypothesis that an eNose can be tuned to the pleasantness scale, and then used to predict the pleasantness of novel odors.

## Results

### eNose training

**Figure 1:**
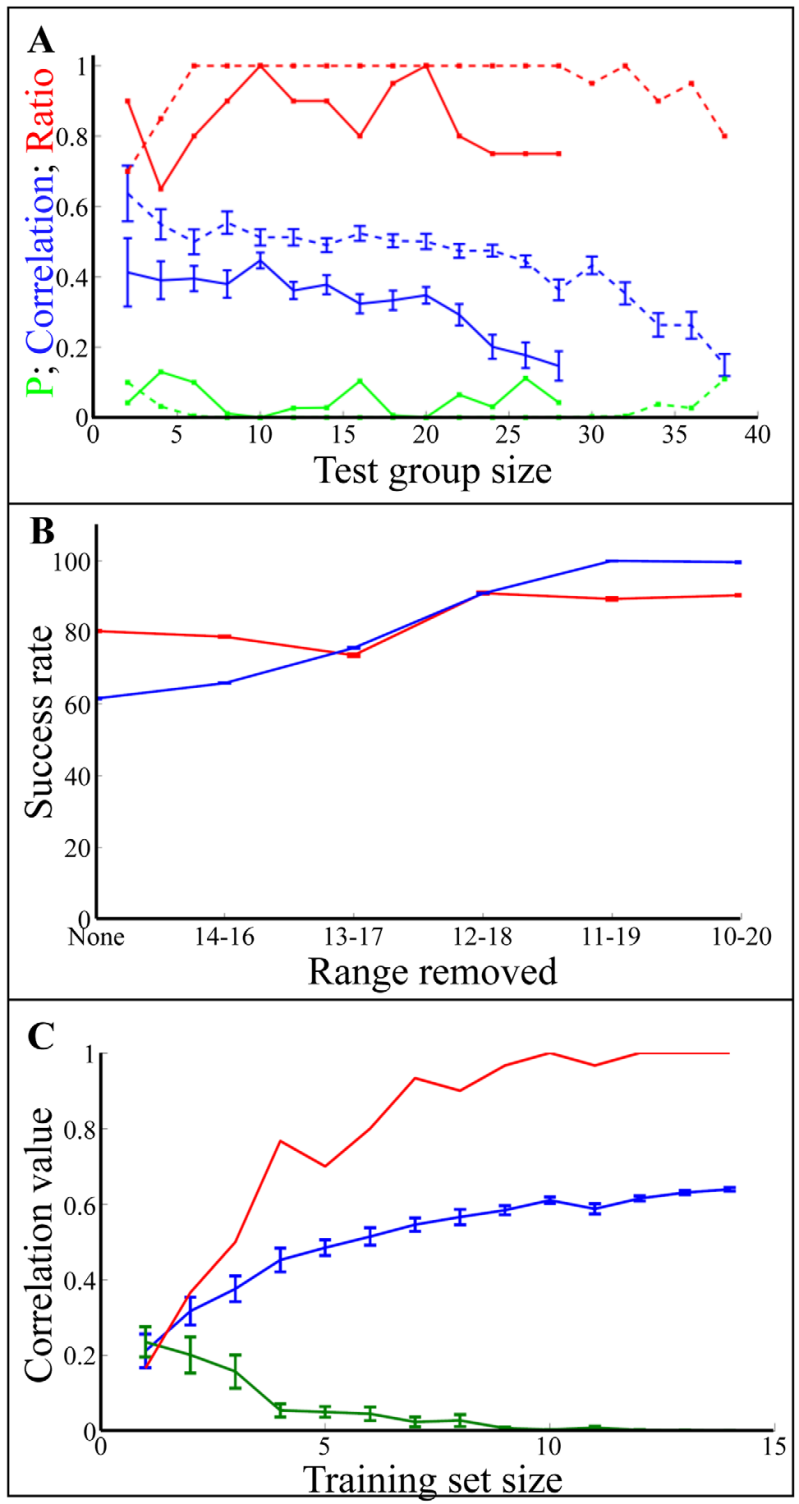
Predicting odor pleasantness with an eNose. **A.** Blue line: correlation values when using different numbers of odorants as test groups and the standard error. The total number of odorants used in this analysis was 76. The numbers in the abscissa are the number of odorants used as a test set. For each point in the graph, we randomly selected odorants and removed them from the training set. We then trained the eNose using the remaining odorants. We repeated this process 20 times for each group size. The red line marks the percent of times the algorithm obtained P<0.05. The green line shows the average P value. Dashed lines show the same analysis but with an initial training set of 98 odorants (the 76 training set plus the 22 essential oils, see text). **B.** The classification success rate as a function of the odors removed from the test set. Odor rates ranged from 0 to 30. We tested the classification rate when we did not remove any odors (None) and when removed an increasing number of odors. For example, 14–16 represent a test in which we did not consider odors with pleasantness ratings ranging from 14 to 16 (e.g. 1 point below and 1 point above the average ratings). Blue line: the essential oils experiment. Red line the second 21 odorants experiment. **C.** Power analysis. Blue: The prediction rate (correlation value) versus the number of odorants used in the training set. Red: the ratio of the number of times the P value was not significant (P>0.05). Green: The mean P value.

We first measured 76 odorants (Supporting Table S1) with a MOSES II eNose. Each odorant was measured on average six times at the same concentration (1ml of pure odorant), providing 424 samples overall. The MOSES II eNose uses 16 different sensors. For each odorant, we extracted 120 features out of the 16 signals (see Methods). Of the 424 samples, 46 signals failed to classify to any of the six repetitions and were removed from further analysis (these failures are the result of the MOSES II device instability). Thus, the eNose measurements resulted in a matrix of 378×120 (424-32 = 378). To prevent excessive influence of one sensor over the others, and to minimize the influence of differences in odorant vapor concentration that can vary despite equal liquid concentration [28], we normalized the columns and rows of this matrix. We then asked human subjects (14–20 per odorant) to rate the pleasantness of each odorant stimuli twice using a visual-analogue scale (VAS) (here the odorants were first individually diluted to create iso-intense perception). Using a training set and test set scheme, we trained a neural network algorithm to predict the median pleasantness of the test set. For a test set of 25 odorants, the median correlation between the eNose prediction and the human rating was 0.46 (average P<0.001, and P<0.05 in 100% of the 20 runs; Figure 1A).

### The eNose generated human-like odorant pleasantness ratings

**Figure 2:**
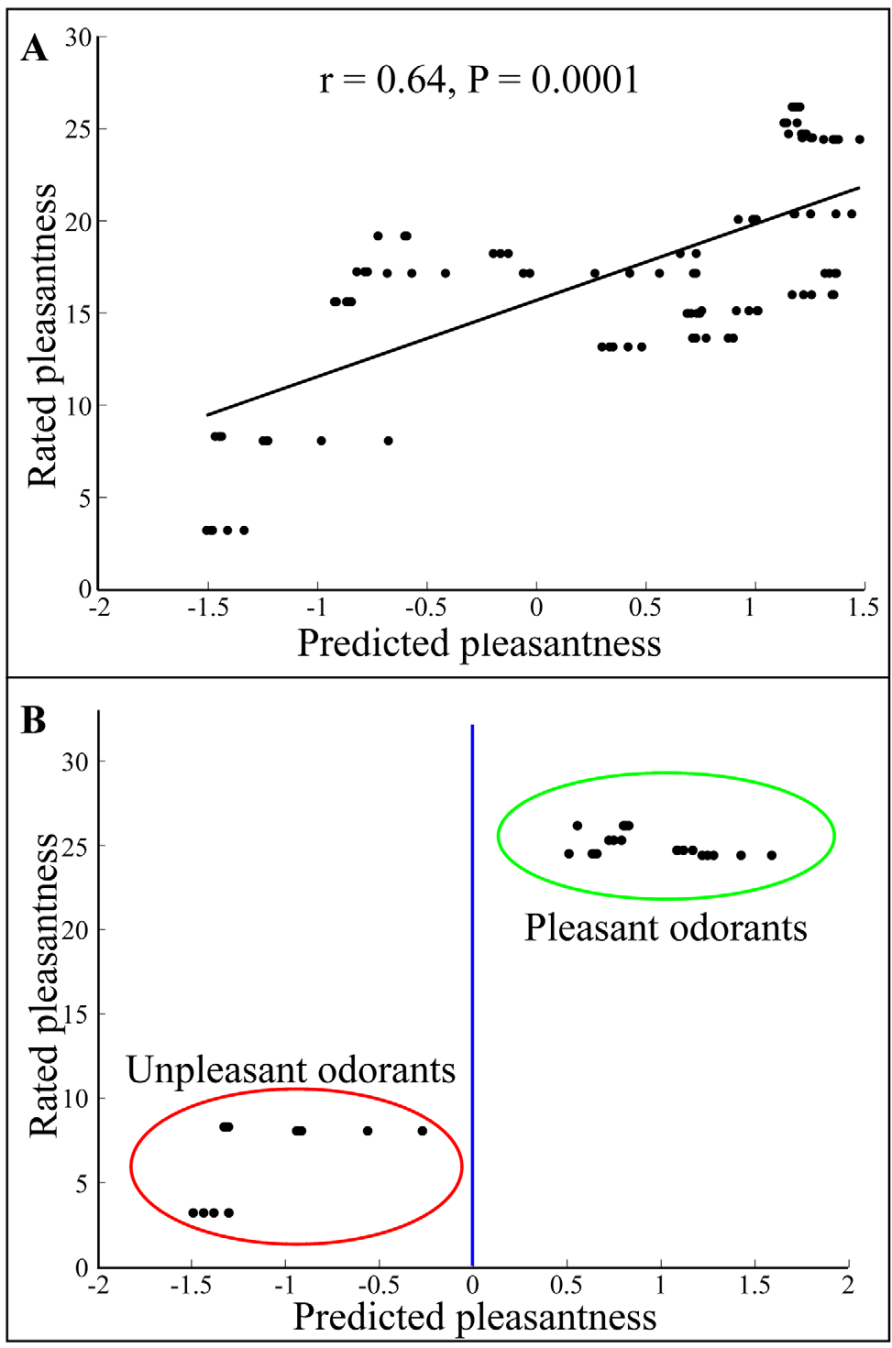
Predicting pleasantness of novel odorants: Essential oils. **A.** The correlation between the eNose pleasantness prediction values of 22 odorant mixtures (essential oils) and the values obtained from human participants. Each dot represents an eNose measurement (many dots overlay) **B.** The result of the classification algorithm when removing all odors with medium pleasantness ratings (below and above 1/3 and 2/3 of the pleasantness scale respectively).

Encouraged by our ability to use an eNose to predict the pleasantness of odorants within the training set (P<0.05 in 100% of the 20 runs), we set out to test its performance with novel odorants, i.e., odorants that were not available during the algorithm development. We used the eNose to measure 22 essential oil odorant mixtures made of unknown components (Supporting Table S1 - essential oils). We measured these oils using the same parameters as in the learning phase, and used the same previously developed algorithm to predict the pleasantness of these odorant mixtures. We then asked 14 human participants to rate twice the pleasantness of these odorants. The average correlation of 30 runs between the machine prediction ratings and the human's median ratings was r = 0.64±0.02 (P<0.0001 in all 30 runs; Figure 2A). We then calculated the correlation between each human's ratings and the median human rating. The correlation was 0.72±0.1, thus the machine-human correlation was 88% (0.64/0.72*100 = 88) of the human to human correlation.

Although these odorants were novel, some of the participants in this study had participated in the original model-building study as well. To address the possibility of any bias introduced by this, we repeated the study again with 17 new participants, and obtained a similar correlation of r = 0.59±0.03, P<0.0001), i.e., a machine-human correlation that was 82% of the human to human correlation.

**Figure 3 pcbi-1000740-g003:**
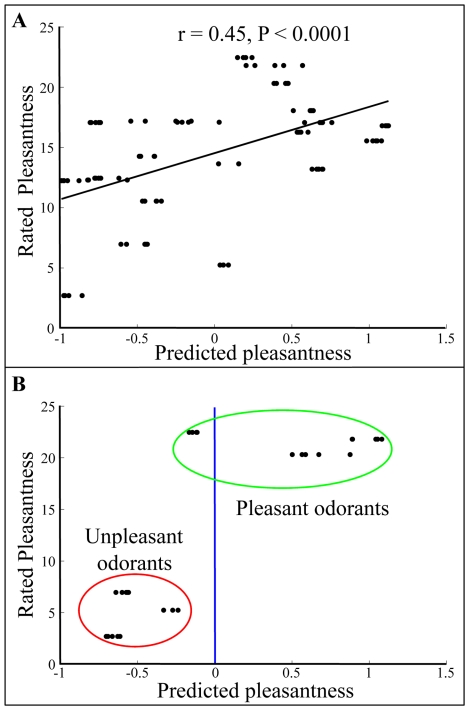
Predicting pleasantness of novel odorants: Neat odorants. **A.** The correlation between the eNose pleasantness prediction values of 21 odorants and the values obtained from human participants. Each dot represents an eNose measurement (many dots overlay) **B.** The result of the classification algorithm when removing all odorants with medium pleasantness ratings (below and above 1/3 and 2/3 of the pleasantness scale respectively).

To further test the robustness of our findings, we conducted a third test of our apparatus, using yet another set of 21 novel neat odorants (Supporting Table S1 - novel odorants experiment) and a group of 18 new participants. In this case, the human to human group average correlation was 0.55±0.18, and the machine-human correlation was r = 0.45±0.02 (P<0.0001 in all 10 runs; Figure 3A). In other words, the machine-human correlation was again 82% of the human to human correlation. We conclude that the eNose generated human-like odor pleasantness ratings.

Up to this point, we considered a continuous scale of odorant pleasantness. Naturally, the correlation between individual human subjects, as well as between human subjects and machine, was lower for ambiguous or intermediately rated odorants. Therefore, we now set out to ask how the eNose would perform if we restricted our analysis to the categorically pleasant and unpleasant odors.

**Figure 4 pcbi-1000740-g004:**
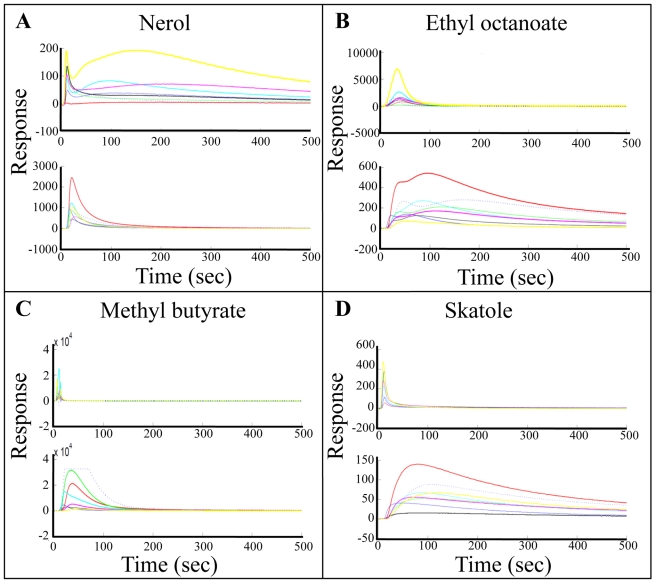
Raw eNose signal amplitude did not reflect pleasantness. Four typical odorant eNose signals of both the QMB sensor module (upper panels) and MOX sensor modules (lower panels). Each line shows the dynamic response of one sensor. Note that both pleasant and unpleasant odorants generated both strong and weak responses. **A** and **B**. An example of two very pleasant odorants. **C** and **D** An example of two very unpleasant odorants.

We conducted a classification analysis after removing odorants with intermediate pleasantness scores (odorants with pleasantness rating ranging from 10 to 20 on the 30 point scale). We classified odorants as pleasant if their predicted pleasantness value was above zero, and unpleasant otherwise. Strikingly, the eNose discriminated between the two odor groups with 99% accuracy (Figure 1B, blue line and Figure 2B). We repeated this analysis on the second set of 21 odorants and 18 participants, and obtained a discrimination success rate of 89% (Figure 1B, red line and Figure 3B). Considering the known relation between odor intensity and odor pleasantness [29]–[31], it is noteworthy that this categorical discrimination of very pleasant from very unpleasant odorants could not have depended on the magnitude of the eNose response alone. This is because the analysis was conducted using the normalized eNose values, and perceptually iso-intense odorants (there was no significant correlation between odor intensity and pleasantness in the two test experiments: P = 0.51 and P = 0.08; |r|<0.35 in both). To reiterate: the odorants were diluted to an equated perceived intensity before their pleasantness was rated by humans. Moreover, examination of the raw eNose response suggested that odorant pleasantness was not a reflection of eNose response magnitude even in the pre-normalized state (Figure 4). We conclude that our apparatus discriminated pleasant odorants from unpleasant odorants, and that this prediction power was not based on odorant intensity.

### Cross-cultural validation

A portion of human olfactory perception is modified through culture [32],[33], context [34], and learning [18]. Although the extent of this portion remains unclear, this nevertheless raises the possibility that the performance of our apparatus was culture-specific. To address this, we set out to test the performance of our apparatus in a group of recent immigrants to Israel from rural Ethiopia. The native Ethiopian participants were adults (mean age = 27) who had arrived in Israel on average 2.3±0.8 years before testing. Because the significant assimilation facing these immigrants in their passage from rural Ethiopia to modern Israel entails a long-term process, this group was all still living together as an independent community in an Israeli Absorption Center where we conducted the experiment. Ethiopian scent-culture is unique in many ways [35], and therefore these participants provided an ideal test for the cultural dependence of our apparatus. Critically, we tested our apparatus with these participants without re-learning or re-calculating any of the apparatus parameters.

Interestingly, despite co-author AM's fluent Amharic, we encountered difficulty in conveying the notion of a visual-analogue rating scale to the native Ethiopian participants. That is, the native Ethiopian participants tended to rate odors at the extremes of the scale, and made lesser use of the middle range. This was made evident in the standard deviation of the VAS scale values. Whereas the average standard deviation of the mean across the same odorants in the native Israeli participants was 6.1±1.5, the average standard deviation of the mean in the native Ethiopian participants was 8±1.5 (T(21) = 5.4, p<0.00002).

**Figure 5:**
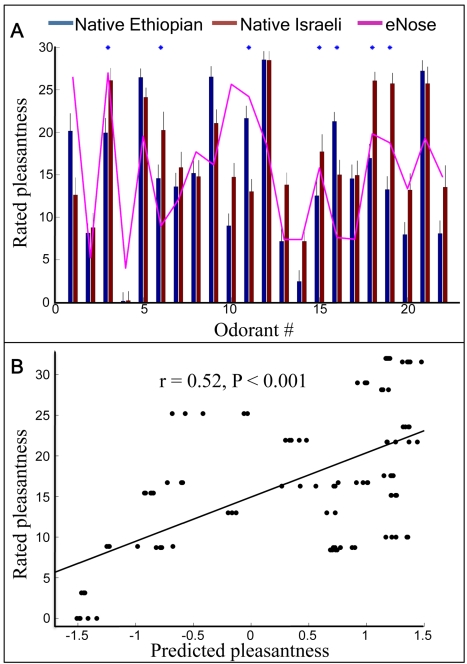
Cross-cultural validation. A. Odorant-specific pleasantness ratings for native Ethiopians (blue), native Israelis (brown), and eNose (pink). The blue stars on the upper x axis denote the 7 odorants where native Ethiopians and native Israelis significantly differed in their pleasantness ratings. Note that for odors #6 #18 and #19 the pink line (eNose) is in fact closer to the native Ethiopians than to the native Israelis even though the eNose was tuned on a separate group of native Israelis. B. The correlation between the eNose pleasantness prediction values of 22 odorant mixtures (essential oils) and the values obtained from native Ethiopians. Each dot represents an eNose measurement (many dots overlay). Comparing Figure 2a to [Fig pcbi-1000740-g003] [Fig pcbi-1000740-g004] 5b reveals that native Israeli participants rated more at the middle of the VAS scale and native Ethiopians rated more at the scale extremes.

The correlation in pleasantness ratings between native Ethiopians and native Israelis was r = 0.75 (p = 0.00004). Although across all odors the median pleasantness assigned by native Ethiopians (14.9±6.5) was not significantly different from the native Israelis (16.7±6.6) (t(21) = 1.8, p = 0.08), when looking at each odorant separately, this group was significantly different from the native Israelis in its pleasantness rating of 7 odorants, 2 of which were rated as significantly more pleasant by native Ethiopians, and 5 of which were rated as significantly less pleasant (Figure 5A). Finally, there was no correlation between the time since arrival in Israel and similarity in rating between the native Ethiopian immigrants and native Israelis (r = −0.17, p = 0.82), suggesting that the native Ethiopian participants remained a homogenous group from the perspective of our question.

The average correlation between the machine prediction ratings and the native Ethiopian's median ratings was r = 0.52±0.01 (P<0.001) (Figure 5B). This correlation was not significantly different from the correlation previously obtained in native Israelis (Fisher z = .69, p = 0.49). Furthermore, the correlation between each native Ethiopian's ratings and the median native Ethiopian rating was 0.60±0.2, thus the machine-human correlation was 86% (0.52/0.60*100 = 86) of the human-to-human correlation in the native Ethiopian population. In other words, the eNose performed equally well across cultures.

Finally, because of the standard deviation in VAS scale usage by the native Ethiopian participants, a classification analysis of extremely pleasant versus extremely unpleasant odorants similar to that conducted in the native Israelis is less informative in this case. Put simply, these participants rated nearly all odorants as extremely pleasant or extremely unpleasant, rendering a classification analysis similar to a simple correlation analysis. Nevertheless, we conducted a classification analysis as well, and the eNose discriminated between the two odor groups with 69% accuracy (p<0.0001).

Because the native Ethiopians and native Israelis significantly differed in their pleasantness ratings for only 7 odorants, this is too small a subgroup for independent statistical analysis. However, a descriptive observation of this subset of odorants remains informative in that for several of the odorants with significant differences, the eNose prediction was in fact closer to the estimates of the native Ethiopians than to the estimates of the native Israelis (e.g., odorants #6,18 and #19 in Figure 5A). This suggests that although the eNose was initially tuned using an independent group of native Israelis, it nevertheless captured a culture-independent aspect of molecular structure that predicts pleasantness.

### eNose algorithm power analysis

To test the dependence of our algorithm on the size of the training set, we repeated the leave-group-out test while augmenting the training set with the essential oils data (Figure 1, dashed blue line). As can be seen in Figure 1, when the training set was larger the prediction accuracy improved. To quantify this relationship, we asked what was the relation between the training set size and the prediction accuracy, or in other words, how many odorants should we present the eNose before we can start predicting? As can be seen in Figure 1C (Blue line) the prediction obtained significance with only 30 samples and saturated with 60–70 samples. Based on this analysis we suggest that around 50 samples are required to predict odor pleasantness with reasonable accuracy using this eNose setup.

To farther test the dependence of our algorithm on the identity of odorants in the training set, we repeated the tests for each of the two novel odorant experiments while augmenting the training data with the other odorant set. The results remained similar: r = 0.56 (P<0.0001) and 100% classification rate in the essential oils experiment and r = 0.49 (P<0.0001) and 88% classification rate in the neat odorants experiment (when removing odorants ranging from 10 to 20 pleasantness ratings). In other words, the prediction was not a result of using a specific training set under specific training parameters.

To further probe the statistical robustness of the results, we scrambled our pleasantness data in a pseudorandom fashion 100 times and repeated our prediction analysis. The average prediction rates dropped to r = 0.08, P>0.23. In other words, the predictions obtained were not due to some internal structure of the data but rather reflected the ability of the algorithm to predict odor pleasantness.

Finally, to ask whether our results were significantly impacted by our outlier removal criteria for eNose measurements, we repeated the correlational analysis using all the data with no exclusions. This resulted in a minimal reduction in correlation between eNose and human pleasantness rating from r = .64 to r = .62, and this correlation remained highly significant (p<0.0004). We also repeated the classification analysis with inclusion of outliers, and classification accuracy remained the same (99%). We conclude that our results were not significantly influenced by outlier removal.

## Discussion

A face can be photographed, digitized and transmitted. Whereas software at the receiving end may be able to rate its beauty in the eyes of previously characterized observers [36], it would not be able to tell us whether a person who's personal preferences were not previously characterized would find beauty in a novel face not part of the learning set. Furthermore, no software can tell us whether a human would like a novel image containing more than faces alone.

Similarly, a musical peace can be recorded, digitized and transmitted. Whereas software at the receiving end may be able to rate the appeal of previously characterized music for novel listeners [37], or the preferences of previously characterized listeners [38] for novel music, it would not be able to tell us whether a person who's personal preferences were not previously characterized would like novel music had they heard it. Furthermore, no software can tell us whether a human would like an auditory recording containing more than music alone.

Here, we eNosed, digitized, and transmitted to receiving software, the smell-print of novel odorants, and in contrast to vision and audition, could predict their pleasantness with accuracy similar to that of a novel smeller. In other words, we could predict whether a person who we never tested before would like the odorant, and this prediction was consistent across Israeli and Ethiopian cultural backgrounds.

We argue that this difference was not a reflection of better hardware (in fact, an eNose is less precise than a modern camera or sound recorder), or better algorithms, but rather a reflection of a fundamental biological property of the sense of smell. These findings imply that unlike in vision and audition, in olfaction pleasantness is written into the molecular properties of the stimulus [17], and is thus better-captured by a machine.

It is tempting to speculate as to the specific molecular aspects that our apparatus was most sensitive to in its determination of pleasantness. For example, careful review of Supporting Table S1 reveals that many low pleasantness odorants were either carboxylic acids or amines, suggesting a functional group specificity. However, other unpleasant odorants, e.g., cyclohexanol, belonged to different functional groups. Previously, we have described a physicochemical odorant axis that corresponds to odorant pleasantness (PC1 of physicochemical structure in Khan et al., 2007). If forced to choose a single verbal label that best describes this axis, one might choose “compactness”, where increased molecular compactness infers reduced odorant pleasantness (Khan et al., 2007). We cannot yet determine, however, whether our apparatus was transducing molecular compactness, or functional group, or some other physicochemical aspect. That said, that the apparatus could nevertheless predict pleasantness across cultures further strengthens the link between odorant pleasantness and odorant structure.

This finding of hard-wired odorant pleasantness is in contrast to the popular notion that odorant pleasantness is both subjective and learned. We argue that in this respect olfactory pleasantness can be likened to visual color. Most would agree that color is hard-wired to wavelength within a predictable framework. That said, color perception can be influenced by culture [39], context [40], as well as by learning and memory [41]. All this does not detract from the hard-wire link between perceived color and wavelength. Similarly, we argue that olfactory pleasantness is hard-wired to molecular structure. That this link is modified through culture [32], [33], context [34], and learning [18], does not preclude the initial hard-wire aspects of this link, and it is this link that we have captured. Indeed, it is thanks to such hard-wiring that rodents bred for generations in predator-free laboratories are nevertheless averse to the smell of predators [42], human new-borns with no exposure to culture or learning are nevertheless averse to unpleasant odorants [22], [43], and that when tested out of context, odorant pleasantness is relatively constant across cultures as revealed here. To stress this point, we predict that if our odorants were presented to subjects within context, e.g., in foods, than the native Israeli and native Ethiopian participants may have then diverged in their pleasantness ratings. For example, peppermint may be rated as a pleasant smelling food in only one of two cultures. However, both cultures may then find peppermint equally pleasant when presented out of context in a jar. Indeed, many may wonder how the French can like the smell of their cheese. However, it is not that the French think the smell is pleasant per se, they merely think it is a sign of good cheese. To prove the point: the French don't make cheese smelling perfume! In other words, culture influences olfactory hedonics mostly in particular contexts. When out of context, odor pleasantness is less culturally variable, and we argue that it is this context-free component that was captured by our apparatus.

Although our results supported our hypothesis, we would like to clearly state their limitations. First, this manuscript used a rather basic commercially available eNose, and more modern eNose technologies may have performed even better. Thus, here we provided proof-of-concept that an eNose can be tuned to a perceptual axis. Beyond proof-of-concept, we do not claim that this iteration represents the best possible implementation of this concept. Second, odorant pleasantness is related to odorant concentration [29]–[31]. Here we negated this source of variance by using equal concentrations across odorants for the eNose measurements, and equal perceived intensities across raters for the human perception measurements. A better algorithm, however, should account for concentration-dependent shifts in pleasantness. Second, we should note that although our training set generated a statistically significant and robust prediction (average P<0.001), the extent of this correlation was not overwhelming (r = 0.45). In fact, the correlations obtained in the later tests with novel odorants and raters were stronger than those of the training set. This reflected our general approach of caution from over-optimizing at training. Specifically, we did not preselect the training odorants to evenly range hedonic space, and we did not preselect for optimal or “professional” human subjects at training. Doing so may have allowed us to generate even stronger predictions than those obtained here. Indeed, when we increased the training set size (Figure 1A dashed line) the correlation value increased substantially (r = 0.56, P<0.0001). Despite these limitations, our suggested device discriminated very pleasant odorants from very unpleasant odorants with high accuracy in both the novel odorants and odorant mixture experiments. Thus, this suggested apparatus can be used for fast odor screening in the scent industry where current methods entail screening by human panels, and may combine with eNose methods for estimating odor intensity [19] and toxicity [44] in order to make for an automated environmental monitor.

Finally, these results may be considered a building-block for digital communication of smell [45]. Individual smells are often composed of thousands of different molecules, each at a particular ratio. Deciphering the exact composition of such odors is a daunting prospect, and recreating these exact mixtures is currently technically limited. In turn, the direction we point towards here is to decipher the odorant-score along main perceptual axes of smell. Once an odorant is characterized along several key axes, a dispensing machine may be able to generate a stimulus defined by the resultant axes-space, an odorant that even if not identical, would nevertheless generate a similar percept.

## Materials and Methods

### Ethics statement

All subjects participated after signing informed consent to methods approved by the Institutional Review (Helsinki) Committee.

### eNose measurements

The MOSES II eNose we used contains eight metal-oxide (MOX) sensors and eight quartz microbalance (QMB) sensors. MOX and QMB are two very different sensor technologies that together capture many facets of the ligand's nature. The 1ml (without any dilution) samples were put in 20-ml vials in an HP7694 headspace sampler, which heated them to 50°C and injected the headspace content into the MOSESII with a flow rate of 40ml/liter. These parameters maximized the number of chemicals that elicited a strong response. To avoid the problem of conditioning we put a blank vial before every measurement and cleaned the system using steamed air after each run of 22 odors. Each analyte was first introduced into the QMB chamber, whence it flowed through to the 300°C heated MOX chamber. The injection lasted 30 seconds, and was followed by a 20 minute purging stage using clean air. Each chemical was measured five or six times over a period of several days. In total, we performed 424 measurements. Each odorant was measured at the same level of humidity and temperature. Each single measurement consisted of sixteen time-dependent signals, corresponding to the eNose sixteen sensors. All the raw eNose data is available for download as Supporting Dataset S1 and on our website at http://www.weizmann.ac.il/neurobiology/worg/materials.html.

### eNose signal feature extraction methods

From each of the 16 sensor signals we extracted four parameters. These parameters were: the signal max value and latency to max, the time the signal reaches the half max value on the decay part and on the rise part. In many cases the signal max value can change considerably between measurements of the same odorants, however, the relative height of the 8 sensors in each of the two sensor modules was largely maintained. Thus, to capture this behaviour we added to each odorant representation the 28 possible ratios of the 8 MOX signals and 28 ratios of the 8 QMB signals. We thus ended up with 120 features for each odorant. To ask whether this feature extractions method was a good representation of the odorants, we clustered the 424 eNose measurements we had into the 76 odorant classes and tested how many odorants fail to cluster into their odor class. Out of the 424 measurements 85% clustered correctly. We removed the 10% signals that failed to cluster to their class, although this did not change the result signifantly (see text). After this signal removal, we ended up with 3 to 6 repetitions per sample measured. We normalized both the feature values and the odorant signature thus removing bias to specific sensor type and odor concentration respectively.

### Human subjects

Fifty six healthy normosmic native Israeli-born subjects (31 females) ranging in age from 23 to 54 years, and 31 healthy normosmic native Ethiopian-born subjects (24 females) ranging in age from 20 to 37 years, participated in the study. The Ethiopian subjects arrived in Israel between 1 and 5.5 years before testing (mean 2.3). All subjects were paid for participation.

### Odor ratings

The total of 123 odors (the 76 training odors and the 43 test odors) were divided into groups of 20–25 odors each. This grouping reflected the maximal time a human subject will typically consistently rate odors (∼40 minutes, with at least 30 seconds between odorant presentations). All odors were first individually diluted to be perceptually iso-intense. Each group of odors was then rated by 14 to 21 subjects. Each subject ranked the pleasantness and intensity of each odor on a visual analogue scale. The visual scale did not contain any markings or indicators other than the terms “very unpleasant” and “very pleasant” at each end. For purposes of analysis, the VAS was later scored from 0 to 30 as a function of the physical location where the VAS line was crossed (0 = very unpleasant, 30 = very pleasant). Each odor was randomly presented twice to each subject. In total, for each odor we had more than 30 ratings (few subjects did not want to rate for the second time). The pleasantness of an odor was calculated by taking the median of all subject's ratings.

### Between and within odor rating correlations

To estimate human to human ratings we calculated the Pearson correlation between all subject pairs and calculated the average correlation value (n>100). To verify that our results were not biased due to the use of visual analogue scale (VAS) we ran an additional experiment using 21 odorants with 6 subjects using a 7 category rating experiments (categories were: The worse odor you ever smelled, very bad, bad, Ok, good, very good, the best odor you have ever smelled). The between human correlation was similar (r = 0.57 in the category rating experiment versus r = 0.6 in the VAS rating experiment; P<0.01 in both). Overall, when considering all our humans ratings, the human to human correlation was 0.45±0.18 (P<0.01) and human to the human group average correlation was 0.67±0.12 (P<0.01). Calculating the average correlation of each subject first rating to his second rating we obtained r = 0.73±0.15 (P<0.01).

### Modeling

We used MATLAB's implementation of a three layered feed-forward back-propagation neural network with 5 internal neurons and 20 epochs. Changing the number of neurons or epochs in the range of 3–10 and 10–30, respectively, did not change the result. The layers' transfer functions were ‘tansig’ and ‘purelin’. The training function was ‘traingd’. To calculate the prediction we ran the algorithm 20 times and used the average value as our best predictor.

### Classification algorithm

To classify odors we used the same algorithm we used for the prediction. Odors with positive predicted value were classified as pleasant and odors with negative predicted value were classified as unpleasant.

## Supporting Information

Table S1 List of odorants used
(0.04 MB XLS)

Dataset S1 Raw eNose data. The zipped directory contains all the raw eNose data in text files, and a read-me file explaining its structure.
(14.88 MB ZIP)
